# Comparison of Risk Factors During First and Second Wave of COVID-19 in Patients with Autoimmune Rheumatic Diseases (AIRD): Results from KRACC Subset

**DOI:** 10.31138/mjr.20230827.co

**Published:** 2023-08-27

**Authors:** Vikramraj Jain, Vineeta Shobha, Sharath Kumar, Ramya Janardana, Sumithra Selvam

**Affiliations:** 1 Clinical Immunology, Bhagwan Mahaveer Jain Hospital, Bengaluru, Karnataka, India,; 2 Department of Clinical Immunology and Rheumatology, St. John’s Medical College Hospital, Bengaluru, Karnataka, India,; 3 Department of Rheumatology, Optima Arthritis and Rheumatology Clinic, Bengaluru, Karnataka, India,; 4 Division of Epidemiology and Biostatistics, St. John’s Research Institute, St. John’s Medical College Hospital, Bengaluru, Karnataka, India

**Keywords:** autoimmune rheumatic diseases, SARS-CoV-2 infection, second wave, risk factors, comorbidity, immunosuppressive therapy

## Abstract

**Background:**

The differential influence and outcome of various risk factors on occurrence of COVID-19 among patients with autoimmune rheumatic diseases (AIRD) during different COVID-19 peaks is underreported.

**Aim:**

To assess the impact and outcome of conventional risk factors, immunosuppressants, and comorbidities on the risk of COVID-19 among AIRD patients during the first two COVID-19 peaks.

**Design:**

Prospective, non-interventional longitudinal cohort study.

**Methods:**

This is a subset of the KRA COVID19 cohort undertaken during the initial wave of COVID-19 (W1) (Apr–Dec2021); and the 2nd-wave (W2) (Jan–Aug2021). Data collected included description of AIRD subsets, treatment characteristics, comorbidities, and COVID-19 occurrence. Risk factors associated with mortality were analysed. The incidence rate was compared with that of the general population in the same geographic region.

**Results:**

AIRD patients (n=2969) had a higher incidence of COVID-19 in the W2 (7.1%) than in the W1 (1.7%) as compared to the general population (Government bulletin). Age (p<0.01) and duration of AIRD (p<0.001) influenced COVID-19 occurrence in W2 while major disease subsets and immunosuppressants including glucocorticoids did not. The W2 had lower HCQ usage (Adjusted Odds Ratio [AOR]-0.81) and comorbidities like hypertension (AOR −0.54) and pre-existing lung disease (AOR −0.38;0.19–0.75) compared to W1. Older age (1.11) and coexistent diabetes mellitus (AOR 6.74) were independent risk factors associated with mortality in W2.

**Conclusions:**

We report 1.7 times higher occurrence, and no influence of major disease subsets or immunosuppressants including glucocorticoids on COVID-19. Age and diabetes were independent risk factors for mortality.

## KEY POINTS

1. AIRD patients had a higher incidence of COVID-19 in the second wave than in the first wave.
2. Higher age and longer duration of AIRD were associated with COVID-19 occurrence in the second wave.
3. Glucocorticoids did not influence the COVID-19 occurrence in this cohort of AIRD patients.
4. Higher age and diabetes were predictors for COVID-19 related mortality in the 2^nd^ wave.

## INTRODUCTION

Mutations in the SARS-CoV-2 genome have influenced several of its properties, such as transmissibility, higher viral loads, shorter time to higher peak loads, pattern and severity of clinical disease, therapeutic measures, efficacy of vaccines, and requirement for public health and social measures.^1,2^ These have resulted in several distinct COVID-19 peaks across the world. The 2^nd^ peak, largely attributed to the delta variant (B.1.617.2), was associated with higher infectivity, younger population, higher oxygen requirement as well as a higher hospital-isation rates in India.^3^

The occurrence and outcome of COVID-19 for patients suffering from autoimmune rheumatic diseases (AIRDs) treated with immunosuppressant therapies differs from that of the general population.^4–6^ This was well documented during the first wave. Data from our large cohort during the first wave demonstrated that conventional risk factors such as gender, coexistent diabetes mellitus, pre-existing lung disease, and smoking were the major contributing risk factors for COVID-19. Glucocorticoids (GC) in moderate dose (7.5–20mg/day) conferred higher risk while other immunomodulators including hydroxychloroquine have no impact.^7,8^ Similar data is also published from large observational, population-based AIRD cohorts wherein no impact of immunosuppressants was observed for mortality outcome.^9^ Apart from glucocorticoid exposure of ≥10 mg/day, none of the DMARDs exposure was associated with higher odds of hospitalisation in the GRA cohort.^4^ However, there are sparse reports worldwide comparing the occurrence, risk factors, and outcomes of AIRD patients between the COVID-19 waves.

Here we report the influence of conventional risk factors, immunosuppressants including GC, and comorbidities on the risk of development of COVID-19 among patients with AIRD from Karnataka, India during the 2nd peak and their outcomes. We also compare the observations to data obtained from the same population during the 1^st^ wave of the COVID-19 pandemic.^7,8^

## METHODS

### Study design and time period

This is an extension of a prospective, non-interventional longitudinal cohort study conducted across 15 referral rheumatology centres undertaken during the initial wave of COVID-19 (W1) across south India viz Karnataka Rheumatology Association COVID Cohort (KRACC). Three of the 15 KRACC centres contributed data on AIRD patients during both the waves of COVID-19; the 2^nd^ wave (W2) (Jan–August 2021) which corresponds to the emergence of the Delta variant and the 1^st^ wave (W1) (up to Dec 2020). Patients who developed COVID-19 during W1 were not included in the controls.

### Data collection and study population

Patients were enrolled into the cohort starting in April 2020 during the W1 and followed up at 1, 3, and 6 months. Follow-up of the cohort was extended to all 15 centres; however, 3 centres contributed data for W2. This subset of patients was contacted telephonically to assess the variables using a structured case report form which included a description of AIRD subsets, treatment characteristics, comorbidities, and COVID-19 infection status. Non-immune mediated rheumatologic disorders and those not on treatment with immunomodulatory therapy were excluded. The data was recorded by trained tele-callers (rheumatology nurses/physician assistants). The diagnosis of COVID-19 was as per Reverse Transcription-Polymerase Chain Reaction (RT-PCR) or Rapid Antigen Test (RAT). COVID-19 testing protocols for symptomatic infection or exposed contacts were as per Government of India recommendations. Ethics approval was obtained at all the participating centres. Waiver of consent was obtained for this telephonic study.

### Statistical Methods

Descriptive statistics were reported as mean with standard deviation, number, and percentages. Factors associated with COVID-19 infection in W1 and W2 were compared to controls (AIRD patients who never contracted COVID-19) using the chi-square test, and ANOVA / Kruskal Wallis test as appropriate. Multiple comparison tests were performed using Bonferroni or Mann-Whitney U tests. A generalised linear model of log-binomial regression was performed to assess the factors associated with COVID positivity in W2 compared to W1 considering time factor W1 and W2 as fixed effects and adjusted for other covariates. Variables that were significant in the univariate analysis were considered for multivariate analysis. P value less than 5% was considered statistically significant. All the analyses were performed using SPSS version 25.0.

## RESULTS

### Demographics and comorbidities

The three centres contributed to a cohort of 3525 patients during W1. Of these participants, 2969 (84%) were willing to follow-up in Wave 2 of COVID. The proportion of patients who contracted COVID-19 in W2 (7.1%) was significantly greater than W1 (1.7%) (p<0.001). **Table 1** describes the comparison of demographics, clinical characteristics, disease subsets, and mortality between W1, W2, and controls. The mean age of the patients was higher in both W1 and W2 compared to control, with a significant difference noted only in W2 (p<0.01). Compared to W1, a greater proportion of patients in W2 had a significantly higher duration of AIRD (>48 months) (p<0.001) while the presence of pre-existing lung disease was significantly lower in W2 (p<0.01). The proportion of hypertensives were significantly higher in W1, compared to W2 (non-significant difference) and controls (p<0.01).

**Table 1.** Comparison of clinical characteristics during 2 time periods (W1, W2) and non-COVID AIRD patients (controls).

	**COVID 19 Wave 1 (W1) n-60**	**COVID 19 Wave 2 (W2) n-211**	**COVID 19 negative (Control) n-3254**	**p value**

Age(yrs)^2^	46.1 ±14.7	45.2 ± 13.7	42.8 ± 14.3	0.001

**Gender**				
Female	43 (71.7)	160 (75.8)	2558 (78.6)	0.287
Male	17 (28.3)	51 (24.2)	696 (21.4)	

**Duration of AIRD in months**				
1–24	26 (43.3)	56 (26.5)	1384 (42.)	0.001
25–48	15 (25.0)	57 (27.0)	606 (18.6)	
>48^2,3^	19 (31.7)	95 (45.0)	1250 (38.4)	

**Diagnosis**				
RA	28 (46.7)	101 (47.9)	1589 (48.8)	0.946
SLE	10 (16.7)	41 (19.4)	636 (19.5)	0.749
Inflammatory Myositis	2 (3.3)	2 (1.0)	78 (2.4)	0.380
Systemic Sclerosis	5 (8.3)	9 (4.3)	127 (3.9)	0.191
Systemic vasculitis	1 (1.7)	3 (1.4)	110 (3.4)	0.281
PsA	2 (3.3)	13 (6.2)	151 (4.6)	0.435
Sjogren’s^1, 3^	4 (6.7)	2 (0.9)	63 (1.9)	0.015
SpA^2^	4 (6.7)	27 (12.8)	255 (7.8)	0.034
Sarcoidosis	0	5 (2.4)	28 (0.9)	0.250
Behçet’s	1 (1.7)	0	2 (0.1)	-
Others	3 (5.0)	8 (3.8)	209 (6.4)	-

**Comorbidities**				
DM	10 (16.7)	30 (14.2)	357 (11.0)	0.134
HTN^1^	18 (30.0)	46 (21.9)	524 (16.1)	0.001
Pre-existing Lung disease^1,3^	10 (16.7)	8 (3.8)	203 (6.2)	0.001

**Mortality^2^**	3 (5.0)	12 (7.9)	58 (1.8)	0.01

Reported as number (%), age as mean (SD); P value using ANOVA and Chi-square.

1 W1 is significantly different from control;

2 W2 is significantly different from control;

3 Significant difference between W1 and W2.

AIRD: Autoimmune Rheumatic Diseases; DM: Diabetes Mellitus, HTN: Hypertension, RA: Rheumatoid Arthritis; PsA: Psoriatic Arthritis; SpA: Spondyloarthritis; SLE: Systemic Lupus Erythematosus.

### Current Immunomodulatory therapy

**Table 2** describes the pattern of immunomodulators in the three groups (W1, W2, and control). The proportion of patients using glucocorticoids, median dose, and duration of glucocorticoid was comparable between the three groups. Other immunosuppressants were comparable between the three groups except for methotrexate (lower in W2, p=0.002) and sulphasalazine (higher in W2, p=0.001). We noted that the usage of HCQ was lowest in W2 (p<0.001).

**Table 2.** Pattern of immunomodulation and associations with COVID19 cohort in W1, W2 and controls.

**Current Immunomodulators**	**Wave 1 n-60 n (%)**	**Wave 2 N-211 n (%)**	**Control N-3254 n (%)**	**p value**

HCQ use (%)^2,3^	32 (53.3)	70 (33.2)	1671 (51.3)	0.001
Median dose (mg/day) (IQR)	300 (200, 300)	300 (200, 300)	300 (200, 300)	0.245
Duration in months (IQR)	18 (6.0, 60.0)	24 (5.2, 60.0)	18 (6.0, 48.0)	0.438

Glucocorticoids (%)	31 (52.5)	93 (44.1)	1527 (47.0)	0.510
Median dose mg/day (IQR)	5 (5, 15)	5 (5, 8)	5 (5, 10)	0.405
Duration (months)	3.5(2.0,11.0)	4.0 (1.0, 7.5)	5.0 (1.0,10.0)	0.603

Methotrexate^2^	31 (51.6)	99 (49.3)	2001 (61.5)	0.002
Azathioprine	3 (5.0)	9 (4.5)	260 (8.0)	0.152
Mycophenolate	4 (6.6)	14 (7.0)	257 (7.9)	0.868
Cyclophosphamide ^1, 3^	3 (5.0)	1 (0.5)	35 (1.1)	0.009
Leflunomide	13 (21.7)	33 (16.4)	695 (21.3)	0.251
Tacrolimus	1 (1.7)	8 (4.0)	152 (4.7)	0.524
Sulphasalazine^2^	3 (5.0)	24 (11.9)	145 (5.7)	0.001
Rituximab	0	1 (0.5)	0	-
TNFi^1^	3 (5.2)	4 (2.0)	20 (0.6)	0.002
Tofacitinib	0	5 (2.5)	66 (2.6)	-
Baricitinib	1 (1.7)	0	5 (0.2)	-
Apremilast	0	1 (0.5)	18 (0.7)	-
Colchicine	1 (1.7)	0	4 (0.2)	-

1 W1 is significantly different from control;

2 W2 is significantly different from control;

3 Significant difference between W1 and W2.

HCQ: Hydroxychloroquine; TNFi: TNF inhibitors.

### Comparison of risk factors for COVID-19 in W1 and W2

The unadjusted and adjusted relative risk of occurrence of COVID-19 in W2 compared to W1 is presented in **Table 3**. Adjusted for age, gender, and along with variables that were significant in the univariate analysis, the usage of HCQ (AOR-0.75, 95% C.I.-0.61, 0.94), the presence of hypertension (AOR-0.45, 95% C.I.-0.25, 0.77) and pre-existing lung disease (AOR-0.32, 95% C.I.-0.17, 0.63), had lower risk associated with COVID-19 in W2 compared to W1.

**Table 3.** Factors associated with COVID positivity in W2 compared to W1.

	**Unadjusted RR 95% C.I.**	**Adjusted RR 95% C.I.**

**Age(yrs)**	1.02 (0.99, 1.03)	1.01 (0.99, 1.03)

**Gender**		
Female	1.37 (0.77, 2.43)	1.45 (0.81, 2.59)
Male		

**Duration of AIRD in months**		
1–24		
25–48	0.85(0.46, 1.54)	-
>48	1.35(0.71, 2.55)	

**Diagnosis**		
RA	1.11 (0.66, 1.85)	
SLE	1.33(0.65, 2.69)	
Inflammatory	0.67(0.17, 2.71)	
Myositis		
Systemic Sclerosis	0.44 (0.18, 1.97)	
Systemic vasculitis	0.52(0.07, 3.72)	
PsA	2.82(0.39, 20.3)	
Sjogren’s	1.09(0.15, 7.80)	
SpA	5.02(0.69, 36.1)	
Sarcoidosis	0.57(0.08, 4.05)	
Behçet’s	-	
Others	-	

**HCQ** use N(%)	**0.75 (0.61, 0.94)**	0.81 (0.48, 1.37)

**Methotrexate** N(%)	**0.83 (0.67, 1.03)**	-

**Glucocorticoids** use N(%)	1.02 (0.81, 1.27)	-

**Comorbidities**		
DM	1.45 (0.71, 2.92)	-
HTN	**0.45 (0.25, 0.77)**	**0.54 (0.30, 0.95)**
Pre-existing Lung disease	**0.32 (0.17, 0.63)**	**0.38 (0.19, 0.75)**

AIRD: Autoimmune Rheumatic Diseases; DM: Diabetes Mellitus, HTN: Hypertension, RA: Rheumatoid Arthritis; PsA: Psoriatic Arthritis; SLE: Systemic Lupus Erythematosus; SpA: Spondyloarthritis; HCQ: Hydroxychloroquine.

### COVID-19 related mortality (Table 4)

Although the proportion of mortality was higher in both the waves compared to control (p=0.01), a slightly higher proportion of mortality was seen in W2 compared to W1. Adjusted analysis showed that older age (AOR-1.11, 95% C.I. 1.03, 1.18) and the presence of diabetes mellitus (AOR 6.74,95% C.I. 1.43, 31.6) were independent risk factors associated with mortality in W2.

**Table 4.** Factors associated with Mortality (n=12) in W2 COVID-19 population.

	**Unadjusted RR 95% C.I.**	**Adjusted RR 95% C.I.**

**Age(yrs)**	**1.09 (1.04, 1.16)**	**1.11 (1.03, 1.18)**

**Gender**		-
Female	2.18 (0.59, 8.07)	
Male		

**Duration of AIRD in months**		
1–24	-	
25–48	3.57 (0.63, 20.1)	
>48	2.58 (0.49, 15.9)	

**Diagnosis**		
RA	1.39 (0.38, 5.11)	
SLE	1.29 (0.27, 6.00)	

**Glucocorticoids**		
N(%)	1.98 (0.48, 8.14)	-

**Comorbidities**		
DM	**8.75 (2.20, 34.8)**	**6.74 (1.43, 31.6)**
HTN	1.02 (0.20, 5.08)	
Pre-existing Lung disease	3.45 (0.37, 31.5)	

AIRD: Autoimmune Rheumatic Diseases; DM: Diabetes Mellitus, HTN: Hypertension, RA: Rheumatoid Arthritis; SLE: Systemic Lupus Erythematosus.

### COVID-19 vaccination

Data pertaining to vaccination was collected in W2. Overall, 1820 (61.3%) had been vaccinated with at least 1 dose, and 704 (23.7%) of the patients had received both doses till Aug 2021. Forty-one patients (2.25%) contracted COVID-19 after the 1st dose of vaccine with a median duration gap of 40 days (15, 75) from the date of the first dose of vaccine, and 13 patients (1.8%) after both the doses of vaccine.

## DISCUSSION

The second COVID wave struck India in April 2021 with higher morbidity and mortality compared to the W1. In this context, we compared the incidence of COVID-19, its predictors, and mortality in patients with AIRDs from three centres located in Bangalore, Karnataka, India during the W1 and W2 and analysed the factors associated.

### Incidence of COVID-19 (W2 vs W1)

We found a higher incidence of COVID-19 among patients with AIRD in the W2 compared to the W1 (7.1% vs 1.7% respectively).^8^ During our study time period (up to Aug 21, 2021) as per the Govt of Karnataka COVID-19 bulletin (https://covid19.karnataka.gov.in/), the cumulative population infected numbers stood at 2,970,000 which corresponds to the incidence rate of 4.3%. We report an overall higher occurrence of COVID-19 in AIRD patients in Karnataka (1.7 times) than the general population in Karnataka, India. An Italian AIRD cohort also showed a significant increase in COVID-19 incidence during the W2 compared to W1(6% vs 3.5% respectively).^11^ This can be attributed to higher transmission of the delta variant and mirrors the peaks observed in the general population.^3^ COVID restrictions (lockdown) were also instituted early and was centralised in 1^st^ wave in India compared to 2^nd^ wave where it was started much later in the wave and was state-specific. Further, the testing was more easily available and accessible by the W2 compared to the W1. In hindsight, it is also our assumption that the incidence of COVID-19 would have been higher if not for the vaccination drive in India that started in February 2021.

### Age

The mean age of patients who developed COVID-19 was similar in both the waves. However, older age was associated with an increased risk of mortality in W2. Reddy et al. reported the mean age of positive cases to be significantly higher in W2 (46.1 ± 16.8 years) as compared to the W1 (35.1 ± 15.9 years).^12^ The Indian population is approximately two decades younger than our cohort. This may explain the finding of older age having higher mortality in W2 compared to a younger age in the general population.

### AIRD duration and disease subsets

Interestingly the duration of AIRD (>48 months) was significantly higher in the W2 patients compared to the W1. Patients with a longer duration of AIRD are more likely to have certain systemic disease manifestations (eg, ILD in RA), disease-related damage and comorbidities making them more susceptible to infections. Notably, the W2 cohort is the patients from W1 who were followed up and may partly explain the increased duration of AIRD. Rheumatoid arthritis was the most common diagnosis in our cohort followed by SLE in both the waves and controls. The disease subsets did not influence the occurrence of COVID-19 or mortality. This was similar to the GRA physician registry, which also did not show any influence of disease subsets on mortality.^10^

### Immunomodulators

We did not find a significant difference in the pattern of immunomodulation between the two waves. This is comparable to Fasano et al who reported a similar pattern of IS among patients with rheumatic diseases in W1 and W2.^11^ HCQ use was lowest in W2 compared to W1 and controls. HCQ was considered to be useful in the prevention and treatment of COVID-19 during W1. However subsequently evidence to the contrary was shown with concerns over cardiac adverse events with HCQ. Fear of adverse events due to misinformation may explain the lower use of HCQ by AIRD patients in W2. Methotrexate (MTX) use was associated with lower risk in W2 compared to controls which were similar to our findings in another AIRD cohort subset (unpublished data). Some case series and observational studies have also suggested that MTX can reduce susceptibility and severity of COVID-19.^13^

### Comorbidities

Interestingly comorbidities like hypertension and pre-existing lung disease had a lower risk of COVID-19 in the W2 compared to W1. Kerai et al also noted a significantly lesser number of patients with coronary artery disease, hypertension, diabetes, and renal dysfunction during the second wave compared to the first among those admitted to ICU.^14^ This is similar to our findings. We postulate that AIRD patients with pre-existing lung disease may have followed stricter precautions during W2 as accurate information was available regarding COVID-19 transmission and risk groups. Higher infectivity of delta variant across may have also negated the effect of comorbidities on the risk of COVID-19.

### Mortality

The W2 saw higher mortality compared to W1 and controls. In contrast, no significant difference in ICU mortality was reported between the two waves by Kerai et al.^14^ Age and diabetes were independent risk factors for mortality in our study. This is consistent with data from the COVID-19 GRA registry that found much of the risk of poor outcomes was attributable to comorbidities in AIRD patients apart from active disease and certain treatments.^15^ Higher infectivity and overall higher mortality in W2 may explain our findings.

Strengths of our study include longitudinal prospective structured data collection in a real-life setting. The AIRD population subsets are well characterized by undergoing treatment at specialist rheumatology units. Ours is one of the few studies to compare the incidence and risk factors of COVID-19 in AIRDs during the two waves. There are few studies so far that have evaluated the same in other subsets of patients with organ-specific autoimmune diseases, immunodeficiency diseases, organ transplant, etc. Our study has several limitations. We could not capture COVID-19 related severity, hospitalisation, and therapy-related information as the healthcare delivery was severely constrained due to the devastating W2 making data interpretation unreliable. Disease activity measures could not be measured as this was a telephonic survey. Further, our data may not have captured all asymptomatically infected patients. The general population data were assessed from the government websites and not by direct questioning of the non-AIRD population through similar telephonic calls.

## CONCLUSIONS

We report a higher occurrence (7.1%) of COVID-19 in AIRD patients in Karnataka which is 1.7 times that of the general Karnataka population (4.3%) as of Aug 2021. Major disease subsets or immunosuppressants including glucocorticoids did not influence COVID-19 occurrence in AIRD patients in W2. HCQ usage, hypertension, and pre-existing lung disease were less likely to be associated with W2 compared to W1. Age and diabetes were independent risk factors for mortality. These differences could represent differences either in susceptibility to Delta variant versus original/wild variant or the effect of vaccination.

## ABBREVIATIONS

AIRD: Autoimmune Rheumatic diseases
ANOVA: Analysis of Variance
AOR: Adjusted Odds Ratio
COVID-19: Coronavirus Disease 19
DMARDs: Disease-Modifying Antirheumatic Drugs
GC: Glucocorticoids
GRA: Global Rheumatology Alliance
HCQ: Hydroxychloroquine
ICU: Intensive Care Unit
ILD: Interstitial Lung Disease
IS: Immunosuppression
KRACC: Karnataka Rheumatoid Arthritis COVID Cohort
RA: Rheumatoid Arthritis
RAT: Rapid Antigen Test
RT-PCR: Reverse Transcription-Polymerase Chain Reaction
SARS-CoV-2: Severe Acute Respiratory Syndrome Coronavirus 2
SLE: Systemic Lupus Erythematosus
SPSS: Statistical Package for the Social Sciences
